# Supplementary material for: Complexome profiling of the Chlamydomonas psb28 mutant reveals TEF5 as an early PSII assembly factor
Source: Plant Cell. 2025 Mar 17;37(6):koaf055. doi: 10.1093/plcell/koaf055 (PMC12164590; doi:10.1093/plcell/koaf055)
Supplement: koaf055_Supplementary_Data [file koaf055_supplementary_data.zip › psb28_interactive.zip!/plots/intensity/qualityPlots/peptides/EFH7_Cre12.g499350.t1.2.html]

### Cre12.g499350.t1.2

Trivial name: EFH7  
SeqCoverage [median]: 11.50  
Mass: 50.93 kDa  
  
 

| Sequence | MissedCleavage | Mass [Da] | wtIntensity | muIntensity | Score |
| --- | --- | --- | --- | --- | --- |
| AIVPTYPPSESHLDR | 0 | 1680.85 | 0.00 | 753.67 | 41.09 |
| FLADVDLLR | 0 | 1060.59 | 614.37 | 0.00 | 65.25 |
| SGSLDGER | 0 | 819.37 | 0.00 | 529.81 | 79.47 |
| SLVATADVR | 0 | 930.51 | 1037.65 | 2179.29 | 73.25 |
|  |  |  | 1652.0 | 3462.8 |  |
